# Arthroscopic Anterior Cruciate Ligament Transection for Osteoarthritis Induction in Rabbits: A Pilot Feasibility and Characterization Study

**DOI:** 10.3390/medicina62091725

**Published:** 2026-09-08

**Authors:** Alina Otilia Adam, Horea Rares Ciprian Benea, Luciana-Mădălina Gherman, Daniel Oltean-Dan, Dragoș Apostu, Dan Gheban, Adrian Bogdan Tigu, Andrei Ivancuta, Alexandru Cristian Sabo, Andrada Uhl, Maria Crișan

**Affiliations:** 1 Department of Orthopedics and Traumatology, “Iuliu Hatieganu” University of Medicine and Pharmacy, 400132 Cluj-Napoca, Romania; adam.alina.otilia@elearn.umfcluj.ro (A.O.A.); olteandandaniel@yahoo.com (D.O.-D.); contact@drapostu.ro (D.A.); 2 Experimental Centre, “Iuliu Hatieganu” University of Medicine and Pharmacy, 400349 Cluj-Napoca, Romania; luciana.gherman@umfcluj.ro; 3 Department of Pathological Anatomy, “Iuliu Hatieganu” University of Medicine and Pharmacy, 400006 Cluj-Napoca, Romania; 4 Department of Personalized Medicine and Rare Diseases, MEDFUTURE—Institute for Biomedical Research, “Iuliu Hatieganu” University of Medicine and Pharmacy, 400349 Cluj-Napoca, Romania; 5 Department of Histology, “Iuliu Hatieganu” University of Medicine and Pharmacy, 400349 Cluj-Napoca, Romania; maria.crisan@umfcluj.ro

**Keywords:** post-traumatic osteoarthritis, animal model, anterior cruciate ligament transection, arthroscopic, cartilage degeneration

## Abstract

*Background and Objectives*: Traditional open anterior cruciate ligament transection (ACLT) surgical models performed through arthrotomy may introduce capsular trauma and postoperative inflammatory responses that can interfere with the interpretation of post-traumatic osteoarthritis (OA). This study aimed to evaluate the feasibility of a minimally invasive, arthroscopic ACLT rabbit model performed entirely under arthroscopic visualization, without arthrotomy, using a 2.4-mm arthroscope and 2.5-mm shaver, and to provide preliminary structural, histological, synovial, and systemic biochemical characterization of the model for future therapeutic testing. *Materials and Methods*: Bilateral arthroscopic ACLT was performed in three skeletally mature New Zealand rabbits, followed by a supervised 12-week exercise protocol. Joint degeneration was evaluated macroscopically and histopathologically using the standardized Pritzker-Osteoarthritis Research Society International (OARSI) grading system. Collagen matrix remodeling was assessed via Picrosirius Red staining. Local intra-articular catabolism was quantified via Enzyme-Linked Immunosorbent Assay (ELISA) for matrix metallopeptidase 13 (MMP-13) and interleukin-1β (IL-1β) from synovial fluid. Systemic oxidative stress, including lipid peroxidation biomarkers, malondialdehyde (MDA), and nitric oxide (NO), was evaluated in serum. *Results:* The arthroscopic approach induced osteoarthritic changes, with severity varying across the cohort. Histopathology revealed vertical matrix fissures and disruption of the normal columnar chondrocyte organization, yielding a median comprehensive Pritzker-OARSI score of 6 (range 6–10), with horizontal degradation restricted to stage 2. Synovial fluid analysis demonstrated measurable concentrations of MMP-13 (188.77 ± 93.87 ng/mL) and IL-1β (174.93 ± 95.51 pg/mL). Furthermore, serum oxidative stress parameters at the 12-week endpoint included lipid peroxidation (MDA: 1.01 ± 0.03 nmol/mL) and NO levels (82.72 ± 0.32 µmol/L), presented descriptively in the absence of a comparator group. *Conclusions:* This study establishes the first multifaceted pathological baseline of a minimally invasive arthroscopic ACLT rabbit model before therapeutic intervention. The arthroscopic ACLT model produced structural cartilage degeneration alongside measurable intra-articular catabolic and systemic oxidative stress changes, establishing a baseline phenotype and a starting point for future therapeutic studies.

## 1. Introduction

Osteoarthritis (OA) is the most prevalent chronic joint disease and a leading cause of pain and disability worldwide [1]. Despite its high prevalence, effective disease-modifying treatments remain unavailable because OA is usually diagnosed after irreversible structural changes have already developed [2]. Consequently, experimental models capable of reproducing the early pathological events of OA have become essential for understanding disease mechanisms and evaluating novel therapeutic strategies.

Osteoarthritis is characterized by disruption of cartilage and subchondral bone homeostasis, resulting from an imbalance between anabolic repair and catabolic degradation. Under mechanical and metabolic stressors, chondrocytes undergo phenotypic shifts, often aggregating into clonal clusters. This chondrocyte activation promotes the production of pro-inflammatory cytokines and matrix-degrading enzymes, particularly matrix metalloproteinases (MMPs), leading to extracellular matrix degradation, loss of proteoglycans, collagen disorganization, and progressive cartilage destruction [3]. These structural alterations are accompanied by subchondral bone remodeling and synovial inflammation, which together drive disease progression [4].

The increasing interest in disease-modifying therapies, including orthobiologics, cell-based therapies, and tissue-engineered constructs, has highlighted the need for reliable preclinical animal models [5]. Such models provide an experimental platform for investigating OA pathogenesis and for evaluating the safety and efficacy of emerging therapeutic strategies before clinical translation.

Numerous experimental models have been developed to reproduce OA, including spontaneous, genetically modified, chemical, and surgically induced models [5]. Among these, surgical destabilization models remain the most widely used because they reproduce progressive mechanical joint degeneration resembling post-traumatic osteoarthritis. In contrast, chemically induced models rapidly generate cartilage degeneration but may not accurately reproduce the multifactorial pathophysiology of human disease [6].

Among surgical models, anterior cruciate ligament transection (ACLT) is one of the most extensively validated methods for inducing post-traumatic osteoarthritis in rabbits [7]. ACLT generates persistent joint instability, leading to abnormal load distribution, progressive cartilage degeneration, subchondral bone remodeling, and osteophyte formation [8]. However, most published ACLT protocols are performed through open arthrotomy, which introduces additional surgical trauma, intra-articular bleeding, and postoperative inflammation that may interfere with the interpretation of biological therapies [9].

Arthroscopic ACL transection has been proposed as a minimally invasive alternative that minimizes capsular disruption while preserving the biomechanical mechanism responsible for osteoarthritis development. Despite its theoretical advantages, this model has not yet been comprehensively characterized regarding its structural, histological, biochemical, and systemic pathological profile during established osteoarthritis. Compared with previously published open ACLT protocols, the arthroscopic approach is expected to reduce surgical morbidity; however, this hypothesis requires direct comparative studies.

Therefore, this pilot methodological study aimed to evaluate the feasibility of a minimally invasive, arthroscopic ACLT rabbit model and to provide preliminary structural, histological, synovial, and systemic biochemical characterization of the model at 12 weeks after osteoarthritis induction, for future therapeutic testing.

## 2. Materials and Methods

### 2.1. Ethical Approval

All experimental procedures were designed, conducted, and reported in accordance with the ARRIVE 2.0 guidelines and were approved by the Ethics Committee of the “Iuliu Hațieganu” University of Medicine and Pharmacy, Cluj-Napoca (Approval No. 307/13.12.2022) and by the Romanian National Sanitary Veterinary and Food Safety Authority (Approval No. 347/20.01.2023). All procedures complied with Directive 2010/63/EU on the protection of animals used for scientific purposes and with the applicable national regulations. Animal housing and care were performed according to institutional guidelines throughout the study.

### 2.2. Animals

This methodological pilot study included three skeletally mature New Zealand rabbits (*Oryctolagus cuniculus*; 2 male, 1 female), weighing 3.0–4.0 kg, constituting the predefined pilot cohort of a staged experimental research program. Mean operative time was approximately 30 min per rabbit (bilateral procedure). All three rabbits were included based on pre-surgical veterinary health screening confirming the absence of pre-existing musculoskeletal abnormality; no a priori exclusion criteria were defined. No animals, knees, or data points were excluded from the analysis after the start of the study. Animals were individually housed under standardized environmental conditions (20–22 °C, 12-h light/dark cycle) with ad libitum access to water and commercial rabbit chow. Following a one-week acclimatization period, all rabbits underwent bilateral arthroscopic ACLT, resulting in six operated knee joints. No intra-articular treatment was administered and no control group was included in this pilot phase, as the purpose of this study was exclusively to characterize the baseline pathological features of the arthroscopic osteoarthritis model before therapeutic intervention. The experimental unit was the individual animal (*n* = 3), so that the six knees were not treated as independent biological replicates. Because this investigation was designed as a methodological feasibility study, no formal sample size calculation was performed. Instead, the cohort size was selected to correspond to the group size anticipated in subsequent comparative therapeutic studies employing this model, allowing the present baseline dataset to serve as a directly comparable reference for future treatment-group cohorts of equivalent size, consistent with the 3Rs principle of reduction.

### 2.3. Anaesthesia and Perioperative Care

General anesthesia was induced by intramuscular ketamine (35 mg/kg) and xylazine (5 mg/kg). Additional doses were administered when necessary to maintain an adequate surgical plane throughout the procedure. Postoperative analgesia consisted of meloxicam (1 mg/kg, subcutaneously) for three days, while antibiotic prophylaxis was provided with a single perioperative dose of enrofloxacin (5 mg/kg). Animals were monitored daily for wound healing, postoperative pain, infection, and general health status. All rabbits resumed unrestricted weight-bearing within 24–48 h after surgery without postoperative complications.

### 2.4. Arthroscopic Anterior Cruciate Ligament Transection

Bilateral knee arthroscopy and ACLT were performed under a single anaesthetic episode. The surgical site was prepared by clipping the hair over both knee joints and aseptic skin preparation. The animal was positioned in dorsal recumbency with the limb in partial flexion. All procedures were performed by the same experienced arthroscopic surgeon.

Arthroscopy was performed using a 2.4 mm diameter arthroscope (30° viewing angle; Storz, Tuttlingen, Germany) connected to a standard video-arthroscopy tower. Two portals were established for each knee: an anterolateral portal for arthroscope insertion and an anteromedial portal for instrumentation. The suprapatellar recess was distended with sterile isotonic saline solution to achieve adequate visualisation of the joint. Following insertion of the blunt trocar and arthroscopic sleeve through the anterolateral portal, the obturator was replaced with the 2.4 mm arthroscope. A standardized diagnostic arthroscopic evaluation of the joint was performed, including assessment of the medial and lateral compartments, the suprapatellar pouch, the intercondylar notch, the articular cartilage surfaces, the menisci, and the cruciate ligaments, confirming normal baseline joint anatomy prior to ligament transection.

The ACL was identified within the intercondylar notch under direct arthroscopic visualisation and was sequentially transected using a 2.5-mm motorized arthroscopic shaver (Storz, Tuttlingen, Germany). Complete transection was confirmed by direct arthroscopic visualization of both ligament remnants together with a positive anterior drawer test performed under anesthesia. Cartilage surfaces were visually inspected under arthroscopic guidance immediately following ligament transection to assess for instrument-related scuffing; no macroscopically visible instrument-related cartilage injury was identified in any knee. Following the procedure, the portal incisions were routinely closed, and the animals were returned to individual housing for recovery.

### 2.5. Exercise Protocol for Osteoarthritis Induction

Beginning one week after surgery and continuing until the end of the experiment, the animals underwent a supervised free-locomotion exercise protocol designed to promote loading of the destabilised joint and accelerate the development of post-traumatic osteoarthritis. Exercise sessions were conducted once weekly in a dedicated room free of obstructions. During each session, rabbits were released individually and encouraged to hop freely. Each session lasted approximately 10 min under continuous supervision to encourage unrestricted weight-bearing. Animals that were reluctant to mobilise spontaneously were gently encouraged by the experimenter.

### 2.6. Experimental Timeline and Study Groups

The experimental timeline for the induction and evaluation of osteoarthritis was structured as presented in Table 1.

### 2.7. Sacrifice and Tissue Collection

At the 12-week endpoint, peripheral blood samples were collected from the marginal ear vein and immediately centrifuged. The serum was separated and stored at −20 °C until oxidative stress analysis. Animals were subsequently euthanized by an overdose of general anesthesia (by intravenous administration of ketamine and xylazine, each at three times the induction anesthesia dose, via the marginal ear vein). Following euthanasia, synovial fluid (approximately 0.4 mL) was aseptically aspirated from each knee joint using a sterile syringe immediately prior to arthrotomy, as part of the same terminal procedure, and stored at −80 °C until Enzyme-Linked Immunosorbent Assay (ELISA) analysis. The joints were then opened for macroscopic assessment, after which the femoral condyles and tibial plateaus were harvested and fixed in 10% neutral-buffered formalin for histological processing.

### 2.8. Macroscopic Assessment

Following arthrotomy, the femoral condyles and tibial plateaus were examined macroscopically. Cartilage degeneration was independently graded by two observers, masked to specimen identity, using the Modified Outerbridge classification [10,11]. The application of this macroscopic scoring approach in rabbit models is supported by the OARSI recommendations [12]. This grading system evaluates cartilage integrity on a five-point scale (0–4), ranging from macroscopically normal cartilage to complete cartilage loss with exposure of the subchondral bone. In cases of disagreement, a consensus score was established after joint review.

### 2.9. Histological Processing and Scoring

After fixation, osteochondral specimens were decalcified in a hydrochloric acid/formic acid solution (Biodec-R, Bio-Optica, Milan, Italy) for 12 h. Samples were subsequently washed in phosphate-buffered saline (PBS, pH 7.4), embedded in paraffin, and sectioned at a thickness of 5 μm. Sections were stained with hematoxylin and eosin (H&E) to evaluate cartilage architecture, structural integrity, and chondrocyte morphology.

Histological severity was independently assessed by two observers, masked to specimen identity, using the Pritzker-OARSI (Osteoarthritis Research Society International) grading and staging system [13]. Cartilage degeneration was evaluated according to structural grade (0–6), representing lesion depth, and stage (0–4), representing the horizontal extent of cartilage involvement. A composite Pritzker-OARSI score (0–24) was calculated by multiplying grade and stage for each evaluation. To account for the bilateral clustered design and report histological outcomes at the animal level (*n* = 3), the higher (worst) composite score between the two knees of each animal was used to select that knee’s grade, stage, and score as representative of that rabbit.

Adjacent sections were stained with Picrosirius Red (PSR) to evaluate collagen fiber organization within the extracellular matrix under polarized light microscopy as previously described in the literature [14,15]. Because PSR birefringence is influenced by fiber orientation as well as collagen architecture, the analysis was interpreted as an indicator of matrix organization rather than a direct quantitative measurement of specific collagen subtypes. Collagen fibers were classified by birefringence color under polarized light: thick, densely packed fibers appear orange to yellow to red, while thin, loosely packed fibers appear green.

### 2.10. ELISA Analysis of Synovial Fluid

Synovial fluid concentrations of matrix MMP-13 and interleukin-1β (IL-1β) were determined using commercially available rabbit-specific ELISA kits (MMP-13: kit catalog number E-EL-RB2225, detection limit: 3.13~200 ng/mL; IL-1β: kit catalog number E-EL-RB0013, detection limit: 15.63–1000 pg/mL; Elabscience Biotechnology, Houston, TX, USA) according to the manufacturer’s instructions. Prior to ELISA analysis, neat synovial fluid was combined with kit diluent to reach the total sample volume required by the assay procedure. Dilution was corrected for individually per sample during back-calculation after plate reading, so that all reported concentrations reflect values in the original, undiluted synovial fluid. All raw optical density readings fell within the kits’ stated detection ranges. Optical density was measured using a microplate reader, and cytokine concentrations were calculated from standard calibration curves generated for each assay. All measurements were performed in duplicate. Biomarker concentrations were averaged across the two knees of each animal to obtain a single representative value per rabbit. MMP-13 and IL-1β were selected to capture two complementary dimensions of osteoarthritis pathophysiology: IL-1β as an upstream pro-inflammatory mediator of cartilage catabolism, and MMP-13 as a key collagenase implicated in the degradation of type II collagen in osteoarthritic cartilage.

### 2.11. Systemic Oxidative Stress Evaluation

Systemic oxidative stress was evaluated in serum using previously validated spectrophotometric methods [16,17]. Total oxidative status (TOS), and total antioxidant response (TAR) were determined according to established protocols, and the results were expressed in μmol H2O2 equiv./L and mmol Trolox equiv./L, respectively. The oxidative stress index (OSI) was subsequently calculated as the ratio of TOS to TAR [17]. OSI was calculated using the native units of the analyzing laboratory’s validated protocol for TOS and TAR. Lipid peroxidation was assessed by measuring malondialdehyde (MDA) levels (nmol/mL) using the thiobarbituric acid method, whereas total serum thiols (SH) were quantified in μmol/L as indicators of antioxidant capacity. Serum nitric oxide (NO) production was estimated indirectly by measuring the stable end-products, nitrite and nitrate (NOx) using the Griess reaction, with final concentrations expressed as µmol/L. All spectrophotometric determinations were performed in triplicate.

### 2.12. Statistical Analysis

Because this investigation was designed as a methodological pilot study involving a limited number of animals, no formal inferential statistical analyses were performed. Histological analysis is reported as median (range). Biomarker outcomes were summarized using descriptive statistics (mean ± standard deviation, range). Continuous variables are presented descriptively with the objective of characterizing the baseline pathological features of the model rather than testing predefined hypotheses. Descriptive statistics were calculated using SPSS version 20.0 (IBM, Armonk, NY, USA).

## 3. Results

Three skeletally mature rabbits (six operated knees) completed the experimental protocol without perioperative complications. Because this investigation was designed as a methodological pilot study, the results are presented descriptively and are intended to characterize the baseline pathological features of the arthroscopic ACLT model rather than to test predefined hypotheses.

### 3.1. Macroscopic Evaluation of Articular Cartilage

**Figure 1:**
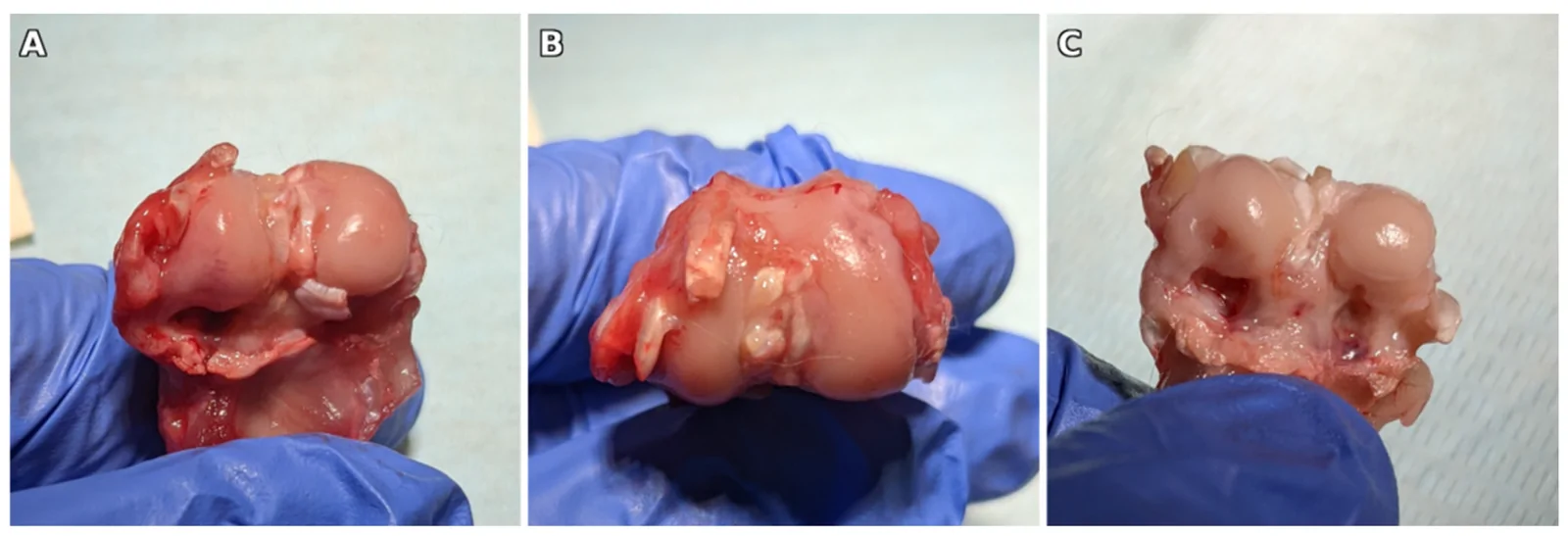
Gross macroscopic appearance of the femoral condyles at 12 weeks post-arthroscopic ACLT: fissures of varying depth (**A**,**B**) and focal full-thickness cartilage defects (**C**).

**Figure 2:**
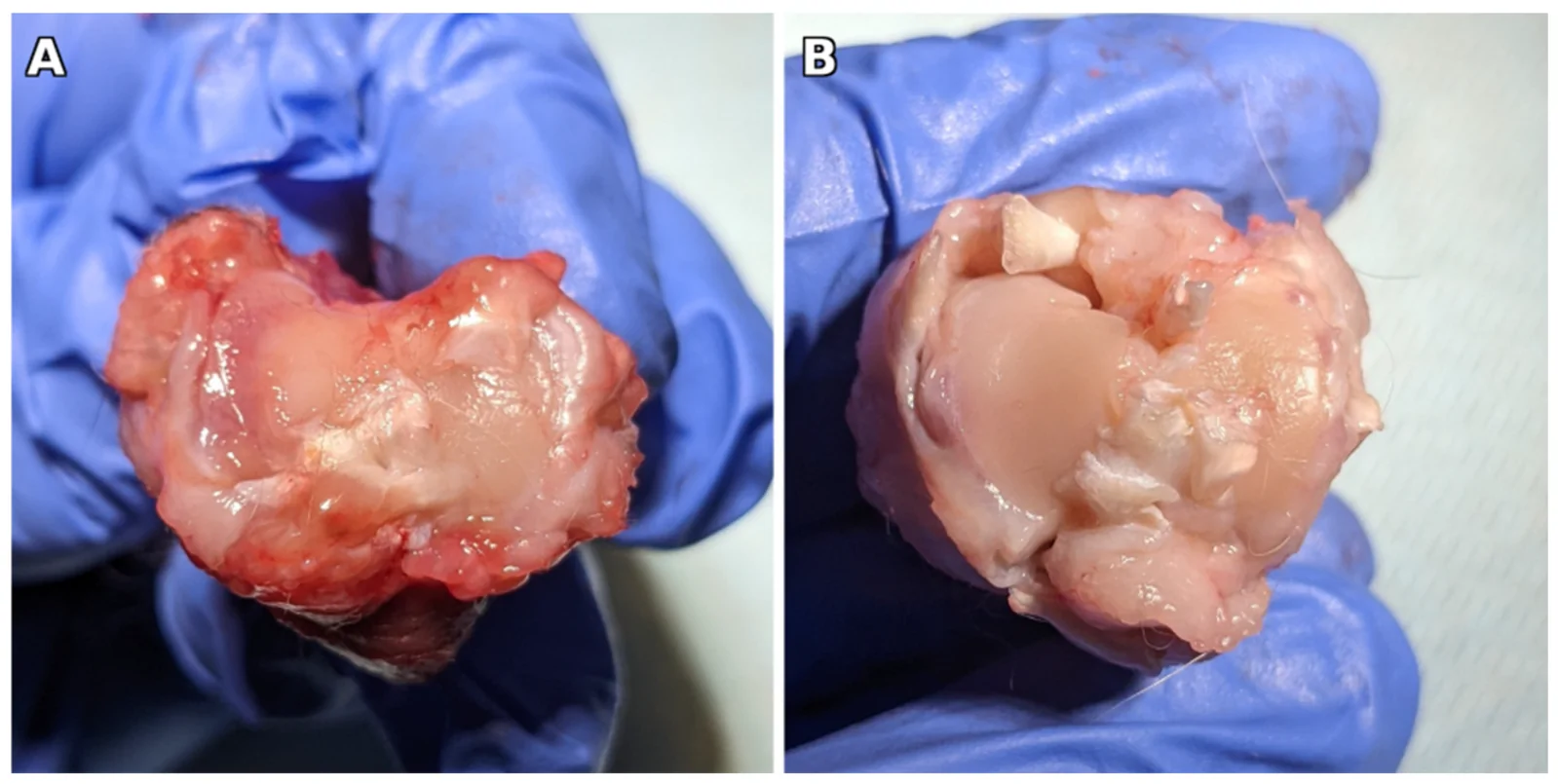
Gross macroscopic appearance of tibial plateaus at 12 weeks post-arthroscopic ACLT: peripheral osteophyte formation (**A**), loss of surface gloss, fibrillation, irregular surface contours (**B**).

At 12 weeks following arthroscopic ACLT, all operated knees exhibited macroscopic changes compatible with osteoarthritis (Figure 1 and Figure 2). The tibial plateaus demonstrated loss of surface gloss, fibrillation, focal cartilage erosion, and irregular surface contours. Corresponding lesions were observed on the femoral condyles, predominantly within the medial weight-bearing regions, where fissures of varying depth and focal full-thickness cartilage defects were identified. Peripheral osteophyte formation and synovial hypertrophy were consistently observed in all joints.

According to the Modified Outerbridge classification, the femoral condyles consistently demonstrated the highest degree of cartilage damage, with lesions corresponding predominantly to grades 3–4. Tibial plateau lesions were generally less severe, ranging from grades 2–3 across all specimens (Table 2).

### 3.2. Histological Assessment of Osteoarthritis Severity, Biochemical Analysis of Synovial Fluid and Systemic Oxidative Stress Evaluation

At the rabbit level (*n* = 3), histological severity was moderate to severe, with a median grade of 3 (range, 3–5), a median stage of 2 (range, 2–2), and a median composite score of 6 (range, 6–10).

The biochemical and systemic oxidative stress parameters are summarized in Table 3. Intra-assay coefficients of variation (CV), calculated from duplicate wells, ranged from 0.40% to 7.91% (mean 5.02%) for IL-1β and 0.62% to 7.23% (mean 4.69%) for MMP-13 across all synovial measurements, within the manufacturer’s acceptable precision range. For comparison with studies employing unit-harmonized conventions, mean OSI calculated after converting TAR to matching units (µmol Trolox equiv./L) was 0.00378 (or 0.378 ± 0.007 using the ×100 scaling convention applied in some prior reports [18]).

**Figure 3:**
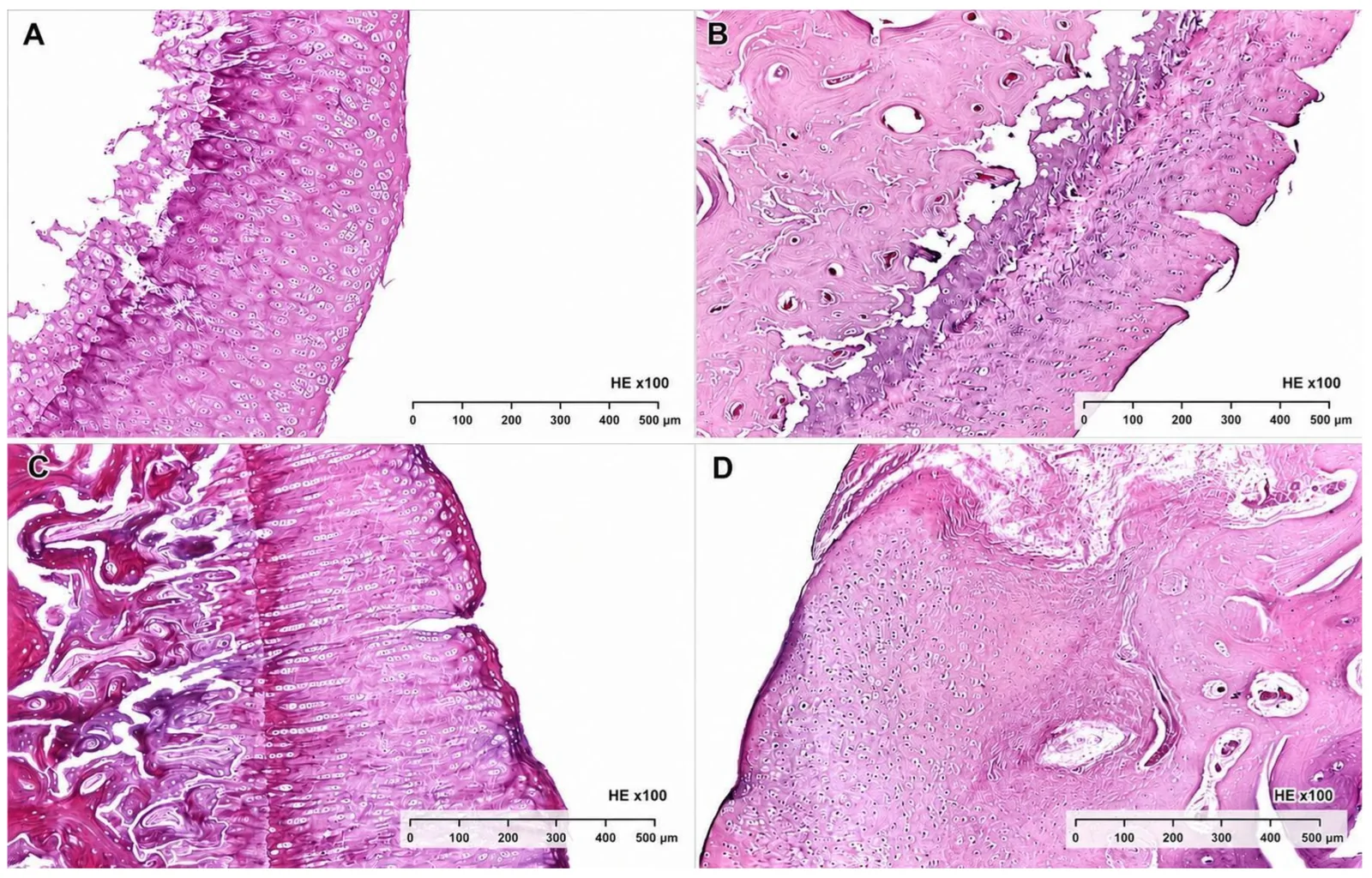
Representative histological sections (H&E) of ACLT-operated knee articular cartilage and subchondral bone representing superficial fibrillation (**A**), vertical fissures (**B**,**C**), and complete ulceration (**D**).

Histological examination demonstrated progressive osteoarthritic cartilage degeneration in all operated joints (Figure 3). Microscopic changes progressed from superficial fibrillation and disruption of the lamina splendens in mild lesions to deep vertical fissures, cartilage delamination, and complete ulceration in advanced specimens. In the most severely affected joints, cartilage loss extended into the calcified cartilage and subchondral bone. Cartilage degeneration was accompanied by reduced cellularity, disruption of the normal columnar chondrocyte organization, and frequent formation of multicellular chondrocyte clusters. Although lesions extended deeply through the cartilage, horizontal involvement remained limited to approximately 50% of the articular surface.

**Figure 4:**
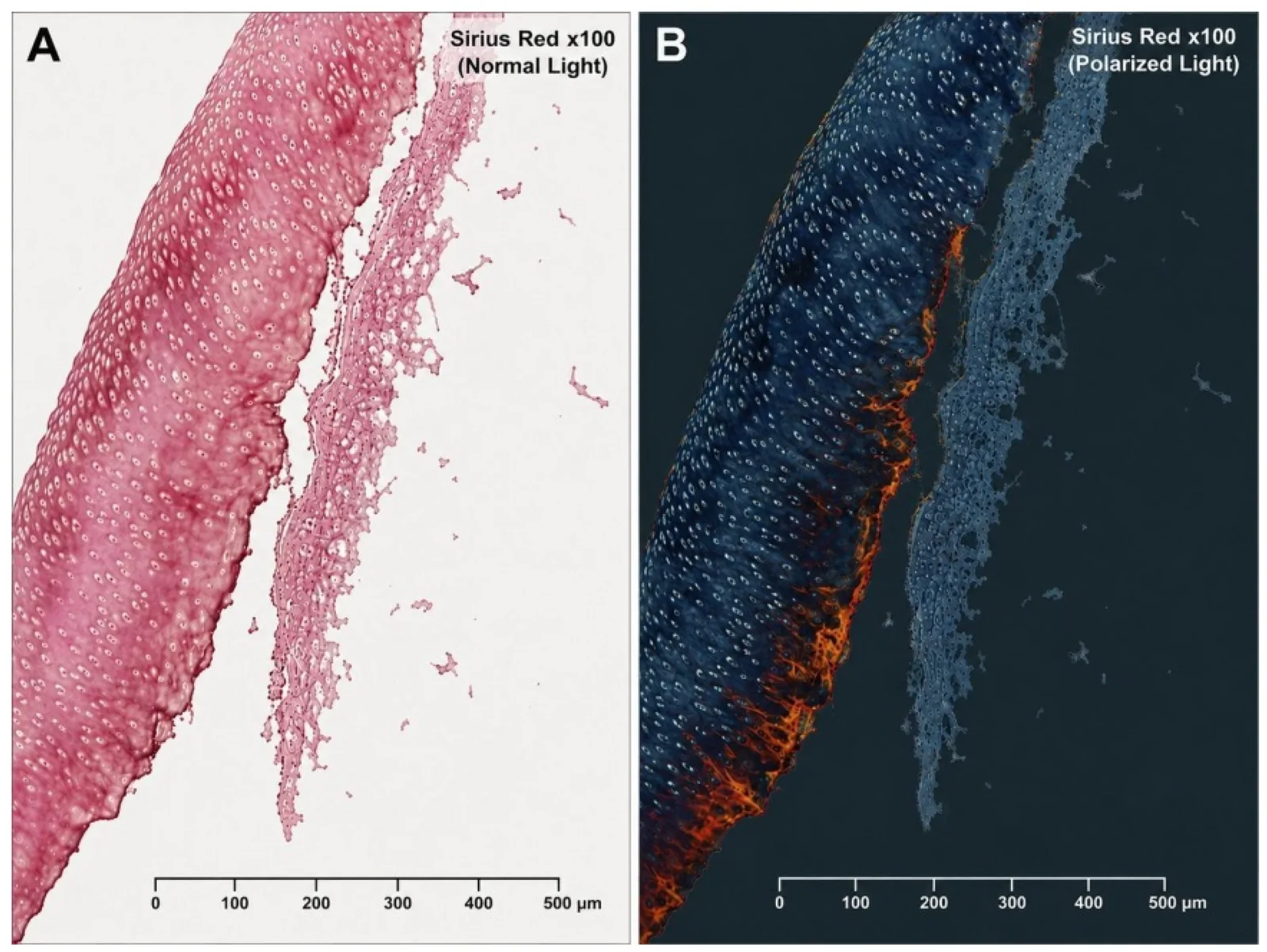
Sirius Red staining of articular cartilage under brightfield (**A**) and polarized light (**B**) microscopy.

Polarized Picrosirius Red analysis demonstrated a predominance of birefringence patterns corresponding to thick, highly organized collagen bundles, whereas signals associated with thinner collagen fibers were limited (Figure 4).

## 4. Discussion

The present pilot study provides a multifaceted characterization of a minimally invasive arthroscopic ACLT rabbit model 12 weeks after osteoarthritis induction. The principal findings were (1) consistent macroscopic and histological cartilage degeneration localized predominantly to the weight-bearing compartments, (2) measurable intra-articular expression of catabolic mediators, including IL-1β and MMP-13, and (3) a baseline systemic oxidative stress profile. Collectively, these findings establish a structural and biological baseline for future therapeutic intervention studies using this experimental model.

Compared with previously published open ACLT protocols, the present arthroscopic approach produced cartilage lesions that developed over a similar timeframe without extensive capsular disruption inherent to arthrotomy [9]. Because no direct comparison with an open procedure was performed, these observations should be interpreted cautiously and primarily in relation to previously published experimental studies.

The macroscopic and histological findings observed in the present study are broadly consistent with previous rabbit ACLT models, in which advanced cartilage degeneration typically develops between 8 and 12 weeks after ligament transection. Yoshioka et al. [19], Papaioannou et al. [20], Huang et al. [21] and Hermeto et al. [22] similarly reported progressive cartilage erosion, osteophyte formation, hypocellularity, and disruption of cartilage architecture during this period. Although the overall OARSI scores in the present cohort were lower than those reported after conventional open ACLT, this observation may reflect differences in surgical approach, disease induction, or methodological assessment rather than reduced osteoarthritis severity alone.

Polarized Picrosirius Red analysis revealed a predominance of highly organized, birefringent collagen bundles within osteoarthritic lesions, indicating altered fibrillar organization relative to the matrix architecture typically observed in native hyaline cartilage. Nevertheless, interpretation of PSR birefringence requires caution because fiber orientation, thickness, and optical alignment substantially influence color distribution under polarized light [15]. Accordingly, the present analysis should be interpreted as reflecting matrix organization rather than absolute collagen subtype composition.

Detection of IL-1β and MMP-13 in synovial fluid indicates an active catabolic and inflammatory intra-articular environment [23,24]. As central drivers of cartilage matrix degradation, the expression of these mediators aligns with the biochemical profile of post-traumatic osteoarthritis described in previous experimental and clinical studies. Joint injury triggers release of damage-associated molecular patterns (DAMPs) that activate synovial macrophages and chondrocytes via nuclear factor kappa-B (NF-κB) signaling, driving IL-1β secretion into the joint space. IL-1β then binds to chondrocyte receptors, activating NF-κB pathways that upregulate MMP-13, the principal collagenase degrading type II collagen. Resulting matrix fragments act as additional DAMPs, sustaining IL-1β production in a self-amplifying loop that drives progressive cartilage breakdown [2,3,25,26].

The severity of molecular breakdown depends heavily on the induction method. Classic surgical models—open ligament transection with meniscectomy [27,28] or medial meniscal root release [29]—typically produce moderate-to-severe OA through acute surgical trauma and mechanical instability. Liu et al. [30] introduced a capsule-sparing medial collateral ligament transection model, in which induced instability partially persisted to six weeks; local catabolic and inflammatory markers rose transiently at two weeks before subsiding. This attenuation might suggest a degree of spontaneous joint stabilization, likely attributable to the robust intrinsic healing capacity of the extra-articular medial collateral ligament. Chemical models, by contrast, achieve comparable severity through intense biochemical rather than mechanical disruption [31].

The present study additionally characterized the systemic oxidative profile associated with untreated arthroscopic ACLT. Although the absence of a healthy control group precludes determination of the magnitude of oxidative imbalance, the obtained values provide an important baseline for future studies investigating antioxidant or disease-modifying therapies.

Systemic oxidative stress parameters were measured at the 12-week endpoint following arthroscopically induced joint degeneration, which may be relevant to growing evidence that osteoarthritis involves metabolic and inflammatory dysregulation beyond the joint itself [32]. The observed antioxidant capacity and NO values are reported descriptively, as the absence of a healthy or sham-operated control group in this pilot cohort precludes interpretation of these findings as evidence of a systemic response or a compensatory mechanism.

For broader clinical context, Gavriilidis et al. [33] reported approximately a 6.5% higher cartilage MDA in knee OA patients than in controls, and Kellgren–Lawrence radiographic severity has been shown to correlate positively with serum free radical and MDA concentrations [34]. However, in the absence of a comparator group, the present findings cannot be directly compared to these previously reported clinical values. Reactive oxygen species are implicated not only in direct tissue damage but also in promoting chondrocyte senescence and a senescence-associated secretory phenotype, upregulating IL-1, IL-6, and MMP expression, while oxidative activation of NF-κB further amplifies MMP production and joint degeneration [32]. This baseline provides an initial systemic metric against which future antioxidant or regenerative therapies may be evaluated in appropriately controlled studies.

This study has several limitations that warrant consideration. First, the small cohort (three rabbits, six knees) and the absence of a non-operated or sham-operated control group and the retention of only consensus (rather than individual pre-consensus) histological scores precluded formal statistical testing and inter-observer agreement calculation; the reported values are therefore descriptive, and the observed inter-joint variability should be interpreted with appropriate caution rather than as evidence of biological heterogeneity per se. We note that in every animal, the two knees showed slightly discordant severity, with one knee modestly more affected than the other; asymmetric within-animal severity despite identical bilateral induction has similarly been reported in other multi-joint OA induction models [35]. We elected to represent each animal by its more severely affected knee, in order to characterize the upper bound of disease severity produced by this induction method. Second, all animals were evaluated at a single terminal timepoint (12 weeks), which characterizes the endpoint pathology but does not capture the trajectory of disease onset and progression—an important consideration given that the model is intended to serve as a baseline for future longitudinal intervention studies. Third, the study design does not allow the individual contributions of joint destabilization versus the supervised exercise protocol to be disentangled; the reported phenotype reflects their combined effect, and it remains unclear how the model would behave under either factor alone. Fourth, OARSI grading was performed on Hematoxylin and Eosin-stained sections rather than on the Safranin-O/Fast Green staining conventionally used to assess proteoglycan depletion in cartilage degeneration models. While this approach has precedent in the literature, it may reduce sensitivity to matrix compositional changes captured by the standard protocol [36]. The comprehensive OARSI scores obtained in this cohort were lower than those reported for traditional open ACLT models [20,21]; while we interpret this as consistent with a more gradual, less traumatic induction process, an alternative explanation—that the arthroscopic technique may under-induce cartilage damage relative to open arthrotomy—cannot be excluded on the basis of this dataset alone and should be addressed in future studies incorporating larger cohorts and intermediate timepoints. Furthermore, the interpretation of the systemic oxidative stress profile is limited by the narrow range of serum total antioxidant response values (coefficient of variation ≈ 0.02%). This tight clustering is a mathematical consequence of the calibration equation’s large additive constant, which can mask the true biological variability among the individual subjects. Finally, the study evaluated structural and biochemical changes at a single terminal endpoint. Consequently, temporal relationships between molecular alterations and cartilage degeneration could not be established.

Despite these limitations, the present study provides the first multifaceted pathological characterization of an arthroscopic ACLT rabbit model combining structural, histological, biochemical, and systemic oxidative stress analyses. These findings establish a robust experimental baseline for future studies evaluating regenerative and disease-modifying therapies under minimally invasive conditions.

## 5. Conclusions

To our knowledge, this is the first study to provide a multifaceted 12-week pathological characterization of a minimally invasive arthroscopic ACLT rabbit model of post-traumatic OA, establishing a baseline phenotype spanning structural cartilage degeneration, intra-articular catabolic activity, and systemic oxidative stress parameters. This baseline provides a structural, biochemical, and systemic reference for future studies of regenerative and disease-modifying osteoarthritis therapies using this model; larger cohorts, longitudinal evaluation, and direct comparison with conventional open ACLT techniques will be needed to further define its translational value.

## Figures and Tables

**Table 1:** Experimental timeline of arthroscopic ACLT induction and osteoarthritis evaluation.

Timepoint	Event	Details
Week 0	Bilateral arthroscopic ACLT	Surgical induction of joint instability
Week 0–1	Initial recovery	Postoperative monitoring; no exercise
Week 1	Exercise protocol initiated	Supervised free-locomotion sessions begin
Week 1–12	Repetitive mechanical loading	Weekly exercise protocol maintained uninterrupted to drive progressive joint degeneration
Week 12	Euthanasia and endpoint evaluation	Macroscopic, histological, biochemical, and systemic characterization

ACLT = anterior cruciate ligament transection.

**Table 2:** Macroscopic evaluation of articular cartilage at 12 weeks post-arthroscopic ACLT (Modified Outerbridge classification).

Category	Parameter	Finding
Femoral condyles	Modified Outerbridge grade	3–4
	Predominant lesion	Erosions (grade 4) and deep, progressive fissuring (grade 3)
	Location	Medial and posterior weight-bearing aspects
Tibial plateaus	Modified Outerbridge grade	2–3
	Predominant lesion	Loss of lustre, fibrillation, pitting, localized cartilage erosion
	Osteophyte formation	Present (peripheral, whitish); observed in all joints
Joint-wide findings	Synovial hyperplasia	Marked; observed in all joints
	Overall osteoarthritic changes	Consistent across all animals

**Table 3:** Summary of histological, biochemical, and systemic oxidative stress findings at 12 weeks post-arthroscopic ACLT.

Category	Parameter	Mean ± SD	Range
Synovial fluid biomarkers (*n* = 3)	MMP-13 (ng/mL)	188.77 ± 93.87	108.44–291.96
	IL-1β (pg/mL)	174.93 ± 95.51	93.45–280.03
Systemic oxidative stress (*n* = 3)	TOS (μmol H_2_O_2_ equiv./L)	4.11 ± 0.07	4.038–4.182
	TAR (mmol Trolox equiv./L)	1.09 ± 0.0002	1.08725–1.08768
	OSI	3.78 ± 0.07	3.712–3.847
	MDA (nmol/mL)	1.01 ± 0.03	0.988–1.040
	SH (μmol/L)	462.67 ± 20.01	443–483
	NO (µmol/L)	82.72 ± 0.32	82.396–83.039

OARSI = Osteoarthritis Research Society International; PSR = Picrosirius Red; MMP-13 = Matrix Metalloproteinase-13; IL-1β = Interleukin-1 Beta; TOS = total oxidative status; TAR = total antioxidant response; OSI = oxidative stress index; MDA = malondialdehyde; SH = total serum thiols; NO = nitric oxide.

## Data Availability

The data are not publicly available until an associated PhD thesis is published. The data presented in this study will be available on request from the corresponding author.

## Abbreviations

The following abbreviations are used in this manuscript:

ACL	Anterior Cruciate Ligament
ACLT	Anterior Cruciate Ligament Transection
DAMPs	Damage-Associated Molecular Patterns
ELISA	Enzyme-Linked Immunosorbent Assay
H&E	Hematoxylin and Eosin
IL-1β	Interleukin-1 Beta
MDA	Malondialdehyde
MMP-13	Matrix Metalloproteinase-13
MMPs	Matrix Metalloproteinases
NF-κB	Nuclear Factor kappa-B
NO	Nitric Oxide
NOx	Nitrite and Nitrate (stable NO end-products)
OA	Osteoarthritis
OARSI	Osteoarthritis Research Society International
OSI	Oxidative Stress Index
PBS	Phosphate-Buffered Saline
PSR	Picrosirius Red
SH	Total Serum Thiols
TAR	Total Antioxidant Response
TOS	Total Oxidative Status
